# Migrant and socioeconomic status might intersect in adverse health outcomes

**DOI:** 10.3389/fpubh.2023.1244612

**Published:** 2023-11-29

**Authors:** David Füller, Stella Juliane Vieth, Bertram Otto

**Affiliations:** ^1^Division of Cardiology, Department of Internal Medicine I, Brandenburg Medical School, University Hospital Brandenburg an der Havel, Brandenburg, Germany; ^2^Brandenburg Medical School (Theodor Fontane), Brandenburg, Germany; ^3^Department of Child and Adolescent Psychiatry, Psychosomatics and Psychotherapy, University Hospital of Würzburg, Würzburg, Germany; ^4^Department for Continuing Education, Graduate School, Evidence-Based Health Care, University of Oxford, Oxford, United Kingdom; ^5^Division of Infectious Diseases, Department of Gastroenterology, Klinikum Ernst von Bergmann, Potsdam, Germany

**Keywords:** migration, social determinants of health, outcomes, intersectionality, socioeconomic factors

European populations show an increasing proportion of immigrants, influenced by conflicts and wars outside EU territory as well as economic factors, among other factors. According to the European Commission's migrant integration statistics, on January 1st 2022, 5.3 % (=23.8 million people) living in the EU-27 were non-EU citizens. Furthermore, there were 13.7 million people (=3.1 %) with the citizenship of one EU member state being permanent residents in another EU-27 country (1). Following this report, foreign citizens, particularly non-EU nationals, on average have lower educational levels, higher rates of unemployment (especially women, irrespective of their level of education), and a significantly increased risk of poverty and social exclusion, even if they are in employment, when compared to national citizens (2). These factors, broadly used in social and medical research, adopted as key social determinants of health (SDoH), and summarized as an individual's socioeconomic status (SES), constitute independent risk factors for poor health and ultimately premature death which are comparable to the mortality risk associated with common widespread disease like hypertension and obesity (3–5).

Evidence on health outcomes among immigrants compared to natives and assessment of exposure-outcome relationships remains limited. Still, health disparities in cancer, diabetes, maternal, child, occupational, and mental health have been reported (6–8). However, differences between countries and regions remain inconclusive, also due to non-uniform or differential assessment of migration backgrounds.

Given the known association of adverse socioeconomic factors and health outcomes, it seems natural to suspect that the higher risk of poor health in a population that is more likely to present with a lower SES can be explained by socioeconomic characteristics. Despite a large body of evidence that describes a higher prevalence and mortality from diseases in the socioeconomically vulnerable, there is few literature specifically assessing the disproportionate burden of poor health in the context of changing demographics (aging population, immigrants and refugees) in Europe.

Individual characteristics and experiences associated with migration can be but are not limited to having faced extreme poverty, high unemployment rates in the home countries associated with low individual job prospects, limited education opportunities, displacement due to war, oppression, discrimination, and persecution, experiencing family separation, having inadequate healthcare access, worse quality of life and living environments, and exposure to the impact of climate change. Conjecturing that the key social determinants of health, including education level, occupational status, and income, have the same impact on migrant health as they do on the health of native citizens ignores these individual characteristics and experiences associated with migration. It overlooks social and cultural determinants affecting health outcomes in a population of immigrants, which also may invalidate traditional SDoH as conventionally defined. However, more research is needed to explore the interplay between well-established socioeconomic factors associated with better health within a broader population, the unique experiences individuals undergo associated with migration (9), and how these experiences impact health outcomes.

## Future directions

Health inequalities within a population are a central challenge of public health. Knowledge how various SDoH drive and determine health outcomes in migrant populations with shared characteristics and how they differ from a population of national citizens is needed.

Intersectionality theory has been tested for self-reported health and disability but not for non-communicable disease incidence and outcomes (10) so it should be tested for disease incidence and mortality in large population-based cohort studies. New studies can be designed to include migrant populations for which detailed sociodemographic and migration-related information is available. Migrant status should distinguish first and next generation immigrants and consider reasons and routes of migration. To estimate SES, a separate analysis of the SES indicators and different SDoH including e.g., housing type or language proficiency can lead to a better understanding of the underlying mechanisms impacting individual health outcomes. This also serves the aim of deriving intervention starting points (11). Moreover, research may reveal factors denoting resilience to adverse experiences that can be forced migration, but also include barriers to healthcare access and discrimination in the host country, and identify social, cultural, economic, and demographic factors that are associated with better health in different populations. Identifying those determinants will enable efforts to reduce the risk for poor health, prevent avoidable medical emergencies and hence, increase healthcare efficacy and population health.

## Author contributions

DF: conceptualization, methodology, investigation, and writing—original draft. SV: conceptualization, investigation, and writing—original draft. BO: conceptualization and writing—review and editing. All authors contributed to the article and approved the submitted version.
